# A Cross-Cultural Analysis of Medicinal Plant Utilization among the Four Ethnic Communities in Northern Regions of Jammu and Kashmir, India

**DOI:** 10.3390/biology11111578

**Published:** 2022-10-27

**Authors:** Tawseef Ahmad Mir, Muatasim Jan, Hammad Ahmad Jan, Rainer W Bussmann, Francesca Sisto, Imad Mohamed Tahir Fadlalla

**Affiliations:** 1 Centre of Research for Ethnobotany, Government Model Science College, Jiwaji University, Gwalior 474009, India; 2 Department of Botany, BFIT Group of Institutions, Dehradun 248007, India; 3 Department of Botany, University of Buner, Swari 19290, Pakistan; 4 Department of Botany, State Museum of Natural History Karlsruhe, 76133 Karlsruhe, Germany; 5 Department of Ethnobotany, Institute of Botany, Ilia State University, Tbilisi 0105, Georgia; 6 Department of Biomedical, Surgery and Dental Sciences, University of Milan, Via C. Pascal 36, 20133 Milano, Italy; 7 Department of Biomedical Sciences, Sudan University of Science and Technology, Khartoum P.O. Box 204, Sudan; 8 Imam Abdulrahman bin Faisal University, Dammam P.O. Box 1982, Saudi Arabia

**Keywords:** ethnomedicine, cross-cultural analysis, four ethnic communities, North Kashmir

## Abstract

**Simple Summary:**

Local ethnic communities have accumulated good traditional ethnomedicinal knowledge on the utilization of plant resources through many generations. In order to preserve and utilize traditional ethnomedicinal knowledge sustainably in the future, ethnobiologists have recently focused on cross-cultural research to record and evaluate the processes driving this system of knowledge evolution within a particular group. The current study records the traditional ethnomedicinal knowledge of plant resources from four ethnic groups in the northern districts of the Union territory of Jammu and Kashmir. A total of 109 plants from 35 families were recorded as being used for the treatment of various disorders by these communities. Asteraceae was found to be the dominant family, with herbs contributing the highest percentage of 86%. The Bakerwal, Gujjar, and Pahadi ethnic groups showed a higher similarity (14% species) in the use of plants, whereas the Bakerwal and Kashmiri ethnic groups used plants with the least similarity (1%). In order to better understand the various traditional plant-use systems, the current study is a collaborative effort that includes not only the documentation but also cross-cultural comparisons of the reported species. This will not only broaden the understanding of cross-cultural ethnobotany in the area but will also create possibilities for locals to benefit from rewards for showcasing their knowledge and taking part in future development projects.

**Abstract:**

Medicinal plants are utilized around the globe for the treatment of a wide range of ailments. This study is an attempt to document the utilization of medicinal plants across the four different cultural groups residing in the rural and remote villages of the northern districts of the Union territory of Jammu and Kashmir, India. To gather information related to medicinal plants and health care practices among the local folk, field surveys were conducted from February 2018 to May 2021. The ethnomedicinal information was gathered through semi-structured interviews and group discussions. During the study, a total of 109 plant species belonging to 35 families were recorded as commonly utilized by the local population, with Asteraceae reported as the dominant family. The most common growth form was herbs, with a percentage contribution of 86%. Leaves (38%) were the most commonly used plant part for the preparation of traditional remedies, and most of the remedies were prepared as paste and applied topically. The highest use value of 0.30 was reported for *Capsella bursa-pastoris*. Greater similarity (14% species) in the usage of plants was shown by Bakerwal, Gujjar, and Pahadi ethnic groups, whereas the least similarity (1%) was observed between Bakerwal and Kashmiri ethnic groups. Based on the results obtained in the present study, further phytochemical and pharmacological analysis of plants is recommended to confirm the efficacy and safety of the remedies used and to possibly elucidate candidates for the development of new drugs.

## 1. Introduction

Indigenous plant medicine is still considered an essential part of healthcare systems across the globe, and traditional medicine comprises both orally transmitted therapeutic methods and codified systems [1]. The use history of medicinally important plants has always been linked with human culture [2]. Of about 350,000–400,000 plant species across the globe, several thousand are utilized to alleviate different disorders [3,4]. According to the World Health Organization (WHO), about 80% of the world’s population still depend on indigenous medicines, and a large population in remote and rural areas uses these medicines as their first line of defense against many ailments [5], especially due to their low cost, acceptability, biomedical benefits and easy accessibility. There is also a growing demand for traditional remedies across the globe [6], and an increasing number of studies on medicinal plants are being published [7].

In India, an estimated 1.5 million healers utilize about 25,000 plant-based traditional remedies. About 6400 flowering plants are believed to have medicinal values, although not more than 10% of these are utilized in modern pharmaceutical industries [8,9,10]. While a variety of studies have been conducted to explore the knowledge associated with traditional healthcare systems of ethnic communities in remote areas of India [11,12], no such detailed report has been published on the cross-cultural utilization of medicinal plants from North Kashmir Himalayas. The northern region of the Kashmir Himalayas, with a total of three districts, including Bandipora, Baramulla, and Kupwara, is a well-characterized part of the greater Himalayas, with a great diversity of flora and fauna [13,14,15]. Most of the populations of these districts reside in rural and remote villages with negligible access to modern healthcare facilities. This study aims to explore the traditional knowledge associated with medicinal plants utilized across the four linguistic ethnic groups, including the Gujjar, Bakerwal, Pahadi, and Kashmiri ethnic groups of North Kashmir. Recent studies have documented the cross-cultural utilization of plant resources, such as in the Balti, Beda, and Brokpa groups in the Trans-Himalayan region of Ladakh and other areas [16]. This research studied how the wild flora of Kashmir Himalaya could improve local life and contribute to the eradication of poverty by providing an in-depth understanding of the ethnomedicinal plant diversity in the region.

According to the recommendation made by the Convention on Biological Diversity [17], local knowledge should be incorporated into future development processes to achieve sustainability because sustainability cannot be attained without taking into account the local knowledge of communities that have a long-standing relationship with their natural resources, including plants. A comprehensive strategy should be used to address the impending extinction problem, as Maffi et al. [18] suggest, to ensure the sustainability of the world. Researchers must concentrate on preserving local and traditional knowledge as a foundation for long-term sustainability in this difficult scenario. In addition to aiding in the protection of traditional knowledge, the field of ethnobiological studies will persuade policymakers to concentrate on the social sustainability of ethnic groups to realize long-term sustainable aims. The current study highlights the historical stratifications and economic standing of the research groups and compares the documented taxa across cultures to comprehend distinct traditional plant usage systems. This will not only increase the region’s understanding of cross-cultural ethnobotany but will also create opportunities for the local population to receive rewards for promoting and celebrating their expertise and participating in future development initiatives. This study focuses on the comprehensive assessment of plant resources with the following objectives: (1) to document the ethnomedicinal uses of the local flora among the different ethnic groups of Kashmir Himalaya, and (2) to make a cross-cultural comparison of the ethnomedicinal uses of the quoted plants.

## 2. Materials and Methods

### 2.1. Study Area

**Figure 1:**
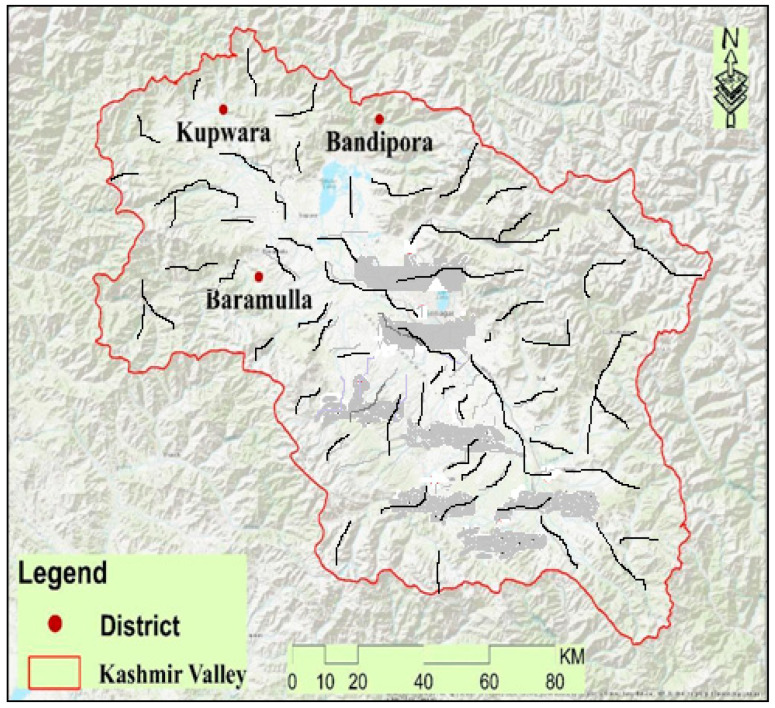
Map highlighting the broader areas surveyed.

Jammu and Kashmir, a former state and now the Union territory of India, stretches over an area of 42,241 km^2^ and has a unique climatic condition and a rich ethnic and phonological diversity. The region is situated to the west of Ladakh, north of Himachal Pradesh, and west of Punjab, and it shares international borders with Pakistan and China to the east. The Jammu and Kashmir state (Jammu, Kashmir, and Ladakh), now a Union territory, has two biogeographic provinces, i.e., Jammu and Kashmir. Geographically, Jammu and Kashmir comprise rugged mountains and barren slopes with climate categories according to the Koppen classification [19]. The main Himalayan range runs along the valley’s northeastern flank. The present study was conducted in the northern region of the Kashmir province (Figure 1). The Kashmir valley has an average elevation of 1850 m above sea level (masl). The broader areas surveyed during the present study included the areas of the districts Bandipora (74°39′ E longitude and 34°25′ N latitude), Baramulla (74°41′ E longitude 34°22′ N latitude), and Kupwara (74°15′ E longitude and 34°01′ N latitude). The region provides a home to different linguistic communities such as Gujjar, Bakerwal, Kashmiri, and Pahadi. The Kashmiri are the descendants of an Indo-European ethnolinguistic group [20], the Pahadi show their descent from the Kash Empire [21], and the Gujjar and Bakerwal are believed to have migrated from Gujrat and the Hazara division of the northwestern frontier province [22]. The region is gifted with rich floral diversity with enormous economic potential. *Fritillaria cirrhosa, Trillium govanianum, Aconitum heterophyllum, Podophyllum hexandrum, Rheum webbianum,* and *Bergenia ciliata* are the important medicinal plants collected by the indigenous population for their livelihood. People of the area have no proper access to modern education services and health care facilities and are thus entirely dependent on locally available medicinal plants for their health care.

### 2.2. Demographic Status of Respondents

To gather the ethnomedicinal information from the study region, a total of 237 informants were selected, with an age group ranging from 18–76 (Table 1). Of the 237 informants, 76 were Gujjar, 51 were Bakerwal, 71 were Kashmiri, and 39 were Pahadi. Most of the informants were in the age group of 56–76 years (41%). Among the interviewed informants, the percentage of illiterate informants was high (67%), and this might be due to the limited educational facilities in the rural and remote villages of the Kashmir valley. A small number of informants had completed their primary and secondary level education. The majority of informants were men (74%), and women comprised 26%. This is because of the cultural norms in which only old-aged women are given access to rituals on any celebration day. The majority of females were not allowed to talk to males outside their community. For these reasons, there was less involvement of women compared to men during the documentation of ethnomedicinal knowledge [23].

### 2.3. Data Collection

To gather information regarding the usage of plants in the study region, field surveys were conducted from February 2018 to May 2021. The data were collected using semi-structured interviews, group discussions, and field observations. Data regarding the human diseases treated, the local names of the plants used, the parts used, the methods of preparation, and the routes of application were gathered during the interviews. Interview questionnaires were prepared in English and then translated into local languages (Gujri, Kashmiri, and Pahadi) (Appendix A). In group discussions, key informants were selected with the help of knowledgeable persons in each village. Special care was taken to avoid non-genuine information [24], and responses were cross-checked through informal methods for confirmation. Consent was always obtained verbally before conducting every interview [10,25]. The project objectives and procedures were clearly explained in the local language to the informants. During field observation, plants, along with their usage, were collected. Much effort was made to collect the plants from their natural habitats in the flowering stage.

### 2.4. Preservation and Taxonomic Verification of Collected Plants

Standard herbarium techniques were used for the collection, drying, mounting, preparation, and preservation of voucher specimens [26]. All the voucher specimens were collected in triplicate, prepared, and then identified with the help of “The Flora of Jammu and Kashmir” [27] and the taxonomists in the field. The botanical nomenclature of the plants was verified using various online platforms (IPNI, Tropicos, and The Plant List). All the identified plant specimens were then verified at the KASH herbarium of the Department of Botany, University of Kashmir, Srinagar, Jammu, and Kashmir, India. The preserved specimens were deposited at the aforementioned herbarium for future reference.

### 2.5. Data Analysis

#### 2.5.1. Overlap Analysis for Cited Plant Species

The ethnomedicinal data of all four communities (Gujjar, Bakerwal, Kashmiri, and Pahadi) were compared. Data are represented in the form of a Venn diagram using the Bioinformatics and Evolutionary Genetics portal (https://bioinformatics.psb.ugent.be/cgi-bin/liste/Venn/calculate_venn.htpl, accessed on 1 April 2021) to illustrate overlaps in the use of taxa.

#### 2.5.2. Use Value (UV)

The use value determines the relative importance of known plant species. In the present study, it was calculated using the following formula [28]:

$$\mathrm{UV}=\sum^{\;}\frac{Ui}{N}$$

where *Ui* is the total number of uses reported by each informant for a given plant species and *N* defines the total number of informants participating in the study. The use value is high when there are many use citations for a plant and vice versa.

## 3. Results and Discussion

### 3.1. Diversity of the Ethnomedicinal Flora

**Figure 2:**
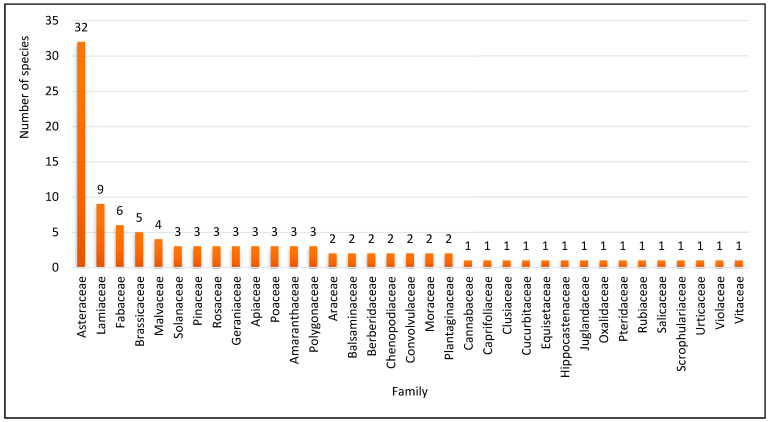
Species contribution of different families.

During the present study, a total of 109 plant species belonging to 35 families were found to be utilized by the people of the study area. Among the reported families, Asteraceae contributed the highest number of species (32 species or 29%), followed by Lamiaceae (9 species or 8%), Fabaceae (6 species or 6%), Brassicaceae (5 species or 5%), Malvaceae (4 species or 4%), and Solanaceae, Pinaceae, Rosaceae, Geraniaceae, Apiaceae, Poaceae, Amaranthaceae and Polygonaceae (3 species or 3% each); all other families contributed less than three species (Figure 2). Likewise, Asteraceae has also been recorded as a dominant family in traditional medicine in other ethnomedicinal studies across India and the rest of the world [29,30,31]. The dominance of this family might be due to its herbaceous life form, extensive distribution, and richness in the study area, and members of this family are well-known for their aromatic quality [32,33]. A large number of species were monotypic, i.e., with one species each, similar to other studies conducted earlier [25,34,35]. Despite their diversity, members of each family are distinguished by their ability to synthesize secondary metabolites with potentially significant biological activity. As a result, they are used in a variety of ways in the traditional healthcare system [36]. For each reported plant species, the botanical name, voucher number, vernacular name, family, habit, part used, preparation, application, ailments treated, and use value were recorded (Table 2). Local people believed that raw materials collected from dense forests or areas less accessible by humans had better efficacy. They, however, often cultivated *Vitis vinifera, Trigonella foenum-graecum, Mentha arvensis, Lavatera cashmiriana, Ficus carica, and Cyndonia oblonga,* among other species, in their gardens since these plant species were hardly available in the wild.

**Figure 3:**
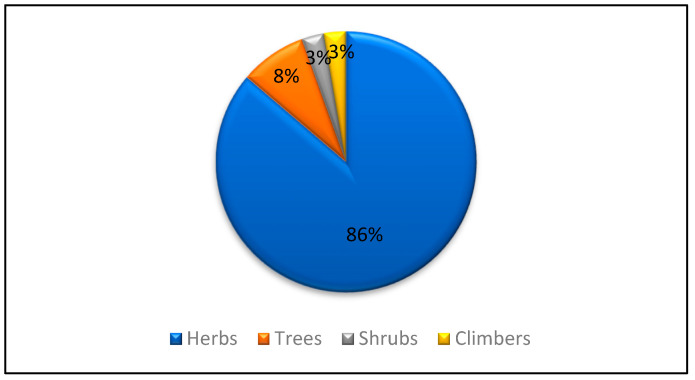
Species contribution of plants according to life form.

Herbs were reported to be the most used life form of the plants (94 species or 86%), followed by trees (9 species or 8%), and climbers and shrubs (3 species or 3% each) (Figure 3). Several other studies from the Kashmir Himalayas and other parts of the world also reported herbs to be the dominant plant species used by local people and practitioners [29,37,38]. The recurrent utilization of herbaceous plants by the local communities of the region can be interpreted to be a result of the rich herb diversity in the environment [39,40]. The people who use medicinal plants in their health care system believe that the materials collected from the deep forests and less human-accessible regions have more curing properties for different types of diseases [11].

### 3.2. Plant Part(s) Used, Mode of Preparation, and Administration

**Figure 4:**
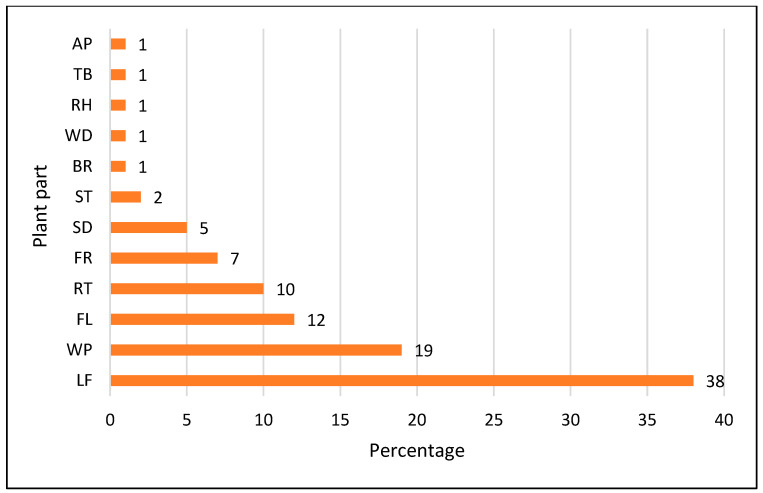
Percentage contribution of plant part used.

**Figure 5:**
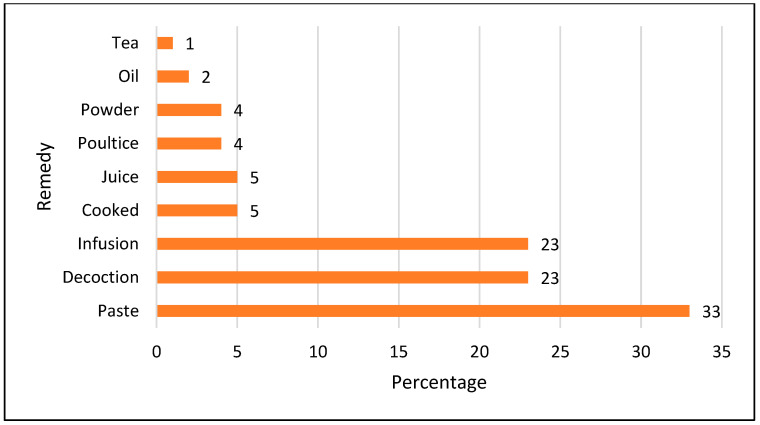
Percentage contribution of herbal remedies.

As far as the utilization of plant parts for the preparation of herbal remedies is concerned, leaves (38%) were the most commonly used plant part, followed by the whole plant (19%), flower (12%), root (10%), fruit (7%), seed (5%), stem (2%), bark, wood, rhizome, tuber and aerial portions (1% each), as shown in Figure 4. Leaves are often used by communities all over the world [41,42,43]. The reason behind this may be that leaves are easy to collect compared to the rest of the plant parts [44] and because, as photosynthetically active parts, the leaves often contain more secondary metabolites [45]. In addition, the difference in plant part consumption could be due to differences in species variety [12]. Most of the remedies were prepared as a paste (33%), followed by decoction and infusion (23% each), cooked and as juice (5%), poultice and powder (4% each), oil (2%), and tea (1%) (Figure 5). The frequent use of decoctions could be due to the perceived high effectiveness in the treatment of a number of diseases or because aqueous extracts are often less toxic than preparations with other extraction methods [46]. Pastes are also commonly used around the globe [39,47]. Most of the herbal remedies were made from a single plant species (monotherapy) rather than by mixing more than one plant species or plant part. Herbal remedies were mostly prepared using fresh plants. These results are in line with other reports from other regions of the world [48,49].

It was found that medicinal plant remedies were administered through oral and topical means by the local population of the region. Topical consumption (52%) was the most commonly used route of administration, followed by oral consumption (48%). The prevalence of topical application is in line with other studies [50,51]. Topical use is considered the most accepted way for the treatment of diseases such as skin disorders, joint pains, wounds, muscular pains, headaches, etc. [52], while oral use is considered ideal for treating internal disorders [52,53]. However, there is a potential difference in the number of doses given to treat a particular disorder.

### 3.3. Cross-Cultural Analysis

**Figure 6:**
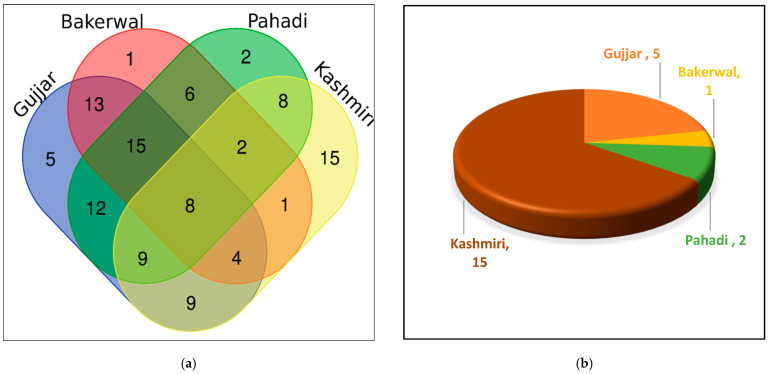
(**a**) Venn diagram showing the overlap of ethnomedicinal usage of plants. (**b**) Plant species uniquely used by different ethnic groups.

A greater similarity (14% species) in the usage of plants was shown by the Bakerwal, Gujjar, and Pahadi ethnic groups, whereas the least similarity (1%) was observed between the Bakerwal and Kashmiri (Figure 6a). The Venn diagram (Figure 6a) shows that fifteen species (14%) were uniquely used by the Kashmiri, while the Bakerwal reported the lowest number of one species (1%). A cross-cultural comparison of plant resources showed that 7% of plants overlapped between the four groups of the study area. The highest number of uniquely used species was used by the Kashmiri community (*n* = 15) in comparison to the Gujjar (*n* = 5), Bakerwal (*n* = 1), and Pahadi (*n* = 2) groups (Figure 6b). The striking diversity in plant use may be attributed to the varied historical stratifications of the investigated groups as well as to distinct sociocultural adaptations and interactions between humans and their environments. These kinds of close similarities in how different tribes use particular plants could be explained by the fact that some of them have engaged in sociocultural agreements with others. For instance, the intermarriage of and similarities in religions, locations, and easy accessibility that the Bakerwal, Gujjar, and Pahadi cultures share; in contrast, the Bakerwal and Kashmiri cultures are distinct from each other, so they exhibit little relationship. The dissemination of ethnobotanical knowledge among them has been influenced as a result. It is also important to note that the fact that there are so many use discrepancies could be related to the fact that the ethnic groups live in such diverse geographic areas. The Pahari and Kashmiri people reside in the middle to upper altitudes, whereas the Bakarwal and Gujjar people inhabit higher elevations. The Bakerwals’ use of mobile pastoralism, which has led to new plant knowledge, is also significant. Haq et al. [16] from the Ladakh region and Aziz et al. [54] from the Pakistan Himalayas conducted a similar cross-cultural analysis and concluded that ethnicity and cultural practices have shaped traditional herbal knowledge among the local inhabitants. Abidin et al. [55] from southwest Pakistan revealed similar findings, which confirm our findings from the Kashmir Himalayan region.

Examining the usage of medicinal plants, all four groups were found to commonly use *Taraxacum officinale* (Handh), *Amaranthus caudatus* (Liss), *Trigonella foenum-graceum* (Meth), *Mentha arvensis* (Pudni), *Cynodon dactylon* (Dramun), *Podophyllum hexandrum* (Wanwangun), *Rosa indica* (Gulab), and *Viola odorata* (Palfort). This overlap might be because these plants are commonly available in the lower as well as higher reaches of the study area or because the informants of all the groups are aware of the medicinal properties of these plants.

In comparison to other groups, *Astragalus grahamianus* (Zand posh) was found to be used only by the Bakerwal tribes. The reason behind this might be that this plant is collected from the upper reaches, along the roadsides, and the same route is used by the Bakerwal tribes for migrating to other places as they are nomadic pastoralists.

Leaves of *Taraxacum officinale* (Handh) are cooked and eaten to treat prolonged menstrual bleeding, weakness, and dyspepsia by all four investigated tribes. Similar results have been reported by Jan et al. [12]. *Daucus carota* (Gazer) is uniquely used by the Kashmiri community. It is due to the presence of the said plant at lower altitudes, where only the Kashmiri people reside. Rhizome infusions of *Acorus calamus* (Vai-gander) are used by the Gujjar and Kashmiri communities as an antispasmodic and an anthelminthic and for the treatment of acidity. Meanwhile, the leaf and flower parts of *Ligularia fischeri* (Gomchwi) are used by Gujjar, Bakerwal, and Pahadi communities but not by Kashmiri. The reason behind this may be the cultural similarities between the three aforementioned groups. Similarly, *Saussurea costus* (Kuth) is also used by Gujar, Bakerwal, and Pahadi ethnic groups. This plant grows commonly in higher reaches, and the Kashmiri communities do not live or hardly live in the upper reaches of the region. This may be the reason behind the use of *Saussurea costus* by only three communities out of the four. *Saussurea costus* is considered a well-known medicinal plant and is commonly utilized for the treatment of many diseases such as asthma, ulcers, inflammatory disorders, stomach problems, and many more [56].

### 3.4. Use Value (UV)

For the evaluation of the local importance of any plant, UV was proposed by Phillips and Gentry [28]. It is not true that medicinal plants with low use values are less important, but it indicates that the knowledge of these medicinal plants is at risk or that there is less availability of the particular medicinal plant [57]. The high UV of medicinal plants in the study region is attributed to their common distribution in the area, and the local people are very familiar with their medicinal uses [58]. The higher the use value, the higher the importance of the particular plant species. However, one cannot distinguish based on UV alone whether a plant is used for single or multiple ailments [59]. In this study, UVs ranged from 0.08 to 0.30, in which the highest value was reported for *Capsella bursa-pastoris* (0.30), followed by *Artemisia absinthium* and *Berberis lycium* (0.26), *Oxalis corniculata* (0.25), and *Juglans regia* and *Saussurea costus* (0.23) (Table 2). Jaradat et al. (2017) also reported *Capsella bursa-pastoris* among the high UV medicinal plants. Bhatia et al. [29] reported *Foeniculum vulgare* among high UV medicinal plants in their study. The lowest UV of 0.08 was recorded for *Amaranthus caudatus, Cosmos bipinatus, cuscuta europea,* and *Impatiens glandulifera*, in contrast to the result reported by Jardat et al. [60].

Meanwhile, *C. bursa-pastoris* has traditionally been used as a medicinal herb to treat vomiting, hemorrhage, conjunctivitis, and hydropsy [61]. Different plant parts of *C. bursa-pastoris* have reportedly been found to contain a variety of biological activities, including those that are anti-tumor [62], anti-inflammatory [63], anti-oxidant [64], anti-microbial [65], and anti-hypertensive [66]. In previous phytochemical studies of *C. bursa-pastoris*, amino acids [67,68], flavonoids [69], alkaloids [70], and essential oils [71,72] were all shown to be present.

## 4. Conclusions

In the present study, it was found that the study area has a rich diversity of medicinally important plant species capable of treating a wide variety of human ailments. It can be concluded from this study that people of the study area possess rich traditional knowledge inherited from their forefathers and that the documentation of this valuable knowledge has provided novel information on the area. Native populations still rely on medicinal plants for their primary health care but, at the same time, are alarmed about the degradation of flora in the wild. It was found that the elderly people possessed a great wealth of indigenous knowledge in comparison to younger ones; this difference in knowledge might be due to the changing lifestyle of the younger generation, the changing views of ethnic communities, and the increasing influence of industrialization, due to which the traditional medicinal knowledge of plant species is vanishing at an alarming rate. Therefore, there is a need to speedily document the important plants and associated knowledge and to take necessary measures for the conservation of these resources to save these treasures; otherwise, a great number of medicinally important plants will become extinct in the wild. To validate this indigenous knowledge, we suggest future phytochemical and pharmacological investigation as these plants may serve for the discovery of new potential drugs.

## Figures and Tables

**Table 1:** Demographic status of respondents from North Kashmir Himalayas.

Demographic Features.	Total	(Linguistic) Ethnic Groups			
		Gujjar	Bakerwal	Pahadi	Kashmiri
**Respondents**	237	76	51	39	71
**Language**		Gujri Urdu	Gujri Pahadi Urdu	Pahadi Urdu	Kashmiri Urdu
**Gender**					
Male	176	56	38	29	53
Female	61	20	13	10	18
**Age range (Years)**					
(Young) 18–28	57	19	14	9	15
(Middle-aged) 29–55	83	27	18	13	25
(Old) 56–76	97	30	19	17	31
**Profession**					
Farmers	29	10	4	3	12
Shepherds	45	9	25	8	3
Semi-skilled workers	46	15	2	10	19
Skilled workers	32	13	4	5	10
Shopkeepers	26	11	2	1	12
Job holders	22	9	2	1	10
Housewives	37	9	12	11	5
Livelihood source		Agriculture and Cattle rearing	Pastoralism	Agriculture and Cattle rearing	Agriculture and Cattle rearing
Descendants of		Northwestern Frontier Province	Migrated from Gujrat	Kash Empire	Indo-European

**Table 2:** Medicinal plants used by the indigenous people of North Kashmir Himalayas.

Family	Botanical Name/Voucher Number	Local Name	Use Recorded across the Cultures				Habit	Part(s) used	Preparation	Application	Ailments Treated	UV
			Gujjar	Bakerwal	Pahadi	Kashmiri						
Amaranthaceae	*Achyranthes aspera* L. 3353-KASH	Phutkunda	Y	Y	N	N	H	LF WP WP	Decoction Paste Infusion	Oral Topical Topical	Dysentery Skin rashes Rheumatism	0.12
	*Amaranthus caudatus* L. 3361-KASH	Liss	Y	Y	Y	Y	H	LF LF SD RT	Decoction Decoction Infusion Decoction	Oral Oral Oral Oral	Diarrhea Dysentery Indigestion Laxative	0.08
	*Amaranthus viridis* L. 3364-KASH	Wazij liss	Y	Y	Y	N	H	LF LF LF	Paste Paste Decoction	Topical Topical Oral	Pimples Joint pain Abdominal pain	0.15
Apiaceae	*Coriandrum sativum* L. 2975-KASH	Daniwal	Y	Y	Y	N	H	WP LF	Decoction Infusion	Topical Oral	Pimples Jaundice	0.19
	*Daucus carota* L. 3390-KASH	Gazer	N	N	N	Y	H	LF LF RT RT	Juice Juice Cooked Cooked	Oral Oral Oral Oral	Anthelminthic Dysentery Fatigue Lactation	0.18
	*Foeniculum vulgare* Mill. 3397-KASH	Badiyan	Y	Y	N	Y	H	FR WP FR FR WP	Decoction Infusion Decoction Decoction Juice	Oral Topical Oral Oral Oral	Colic infection Gum disease Sore throat Urine infection Constipation	0.13
Araceae	*Acorus calamus* L. 3365-KASH	Vai-gander	Y	N	N	Y	H	RH RH RH	Infusion Infusion Infusion	Oral Oral Oral	Antispasmodic Anthelminthic Acidity	0.20
	*Arisaema jacquemontii* Blume. 2968-KASH	Hapet-Gogji	Y	Y	N	N	H	RT LF	Paste Paste	Topical Topical	Blisters Pimples	0.19
Asteraceae	*Achillea millefolium* L. 2966-KASH	Pahel-gaash	N	N	N	Y	H	LF LF	Infusion Infusion	Oral Oral	Stomach pain Dysentery	0.17
	*Anthemis cotula* L. 2967-KASH	Fakh-gassh	Y	N	Y	N	H	FL FL FL FL	Juice Decoction Decoction Infusion	Topical Topical Topical Topical	Skin antiseptic Skin allergy Muscle pain Burns	0.22
	*Arctium lappa* L. 3367-KASH	Phughood	N	N	Y	Y	H	RT RT RT	Paste Paste Paste	Topical Topical Topical	Boils Burns Blisters	0.11
	*Artemisia absinthium* L. 2969-KASH	Tethwan	Y	N	Y	Y	H	LF LF	Infusion Infusion	Oral Oral	Intestinal worms Abdominal pain	0.26
	*Artemisia annua* L. 3368-KASH	Dudh-kandij	N	Y	Y	N	H	LF RT RT	Infusion Infusion Infusion	Oral Oral Oral	Diabetes Intestinal worms Jaundice	0.12
	*Artemisia moorcroftiana* Wall. ex DC 3369-KASH	Jangli-tethwan	Y	Y	Y	N	H	WP WP LF LF	Decoction Decoction Decoction Decoction	Oral Oral Oral Oral	Abdominal pain Gas formation Indigestion Intestinal worms	0.11
	*Artemisia scoparia* Waldst. and Kit. 3370-KASH	Pari-chaw	Y	N	Y	N	H	WP LF LF	Infusion Infusion Decoction	Oral Oral Oral	Inflammation Liver infection Fever	0.14
	*Bidens pilosa* L. 3373-KASH	Kumber	Y	N	Y	N	H	LF LF WP WP	Paste Powder Powder Powder	Topical Topical Topical Topical	Eye pain Stomach ulcer Cold Cough	0.13
	*Bidens tripartita* L. 3374-KASH	Kumber	N	N	N	Y	H	LF WP WP WP	Paste Paste Paste Paste	Topical Topical Topical Topical	Blisters Cough Cold Eye disease	0.11
	*Calendula officinalis* L. 3375-KASH	Hamesh-bahar	N	Y	Y	Y	H	LF FL FL	Paste Paste Paste	Topical Topical Topical	Herpes Boils Burns	0.20
	*Carpesium abrotanoides* L. 3378-KASH	Ban-sario	Y	Y	N	N	H	SD SD	Decoction Decoction	Oral Oral	Intestinal worms Indigestion	0.09
	*Centaurea iberica* Trevir. ex Spreng 3381-KASH	Krech	Y	N	N	Y	H	LF LF LF	Paste Paste Paste	Topical Topical Topical	Skin rashes Burns Wounds	0.20
	*Cichorium intybus* L. 2973-KASH	Kaw-hand	N	Y	Y	N	H	WP WP WP LF LF	Decoction Decoction Decoction Cooked Cooked	Oral Oral Topical Topical Topical	Diarrhea Body weakness Fever Joint pain Fractured bones	0.22
	*Cirsium arvense* (L.) Scop. 2974-KASH		N	Y	Y	N	H	LF FL FL	Paste Paste Paste	Topical Topical Topical	Wounds Headache Joint pain	0.12
	*Conyza bonariensis* (L.) Cronquist 3385(Shashedra)	Shashedra	N	N	Y	Y	H	WP WP LF LF	Infusion Infusion Infusion Infusion	Oral Oral Oral Oral	Painful menstruation Painful urination Kidney infection Anthelminthic	0.14
	*Conyza canadensis* (L.) Cronquist 2982-KASH	Shal-lutt	N	N	N	Y	H	LF RT RT	Paste Infusion Infusion	Topical Oral Oral	Wounds Diarrhea Dysentery	0.12
	*Cosmos bipinnatus* Cav. 3386-KASH	Mazan-posh	Y	N	N	Y	H	FL FL FL	Decoction Decoction Paste	Oral Oral Topical	Jaundice Fever Headache	0.08
	*Cotula anthemoids* L. 3387-KASH	Thol-bobul	Y	Y	N	N	H	WP WP WP WP WP	Decoction Decoction Decoction Infusion Poultice	Topical Topical Topical Topical Topical	Nasal congestion Joint pain Headache Wounds Fractured bones	0.20
	*Galinosoga parviflora* Cav. 2983-KASH	Machawagan-ghass	Y	N	N	N	H	WP WP WP	Poultice Paste Paste	Topical Topical Topical	Joint pain Cuts Wounds	0.14
	*Lactuca saligna* L. 3406-KASH	Dodhkandiej	Y	N	Y	Y	H	WP WP WP WP	Decoction Infusion Infusion Decoction	Topical Oral Oral Oral	Joint pain Diarrhea Dysentery Abdominal pain	0.11
	*Leucanthemum vulgare* Lam. 2990-KASH	-	Y	Y	N	Y	H	LF LF LF	Decoction Decoction Paste	Oral Topical Topical	Cough Burns Wounds	0.17
	*Ligularia fischeri* (Ledeb.) Turcz. 3622-KASH	Gomchwi	Y	Y	Y	N	H	LF LF FL LF	Infusion Paste Paste Infusion	Oral Topical Topical Oral	Jaundice Anti-inflammatory Arthritis Liver infection	0.19
	*Myriactis nepalensis* Less. 3418-KASH		Y	Y	N	N	H	ST ST ST ST	Paste Paste Paste Paste	Topical Topical Topical Topical	Wounds Chapped hands Cracked heels Cracked lips	0.11
	*Saussurea costus* (Falc.) Lipsch. 3442-KASH	Kuth	Y	Y	Y	N	H	RT RT RT RT	Decoction Decoction Decoction Decoction	Oral Oral Oral Oral	Asthma Bronchitis Cough Cold	0.23
	*Senecio chrysanthemoides* DC. 3443-KASH	Bagghu	Y	N	N	Y	H	FL LF LF	Paste Paste Paste	Topical Topical Topical	Wounds Cuts Skin rashes	0.09
	*Sigesbeckia orientalis* L. 3444-KASH		N	N	N	Y	H	LF LF WP	Decoction Decoction Paste	Topical Topical Topical	Joint pain Skin rashes Blisters	0.09
	*Sonchus arvensis* L. 3003-KASH	Dudij	N	Y	Y	N	H	LF LF LF	Decoction Paste Poultice	Topical Topical Topical	Skin rashes Wounds Swelling	0.14
	*Tagetus erecta* L. 3004-KASH	Guttaposh	N	Y	Y	Y	H	FL FL	Infusion Infusion	Oral Oral	Urinary infection Colic infection	0.09
	*Tagetus minuta* L. 3453-KASH	Jalanijafar	Y	N	Y	Y	H	FL FL LF	Infusion Infusion Decoction	Oral Oral Oral	Blood purifier Dyspepsia Fever	0.10
	*Taraxacum officinale* F.H. Wigg. 3005-KASH	Handh	Y	Y	Y	Y	H	LF LF LF	Cooked Cooked Cooked	Oral Oral Oral	Prolonged menstrual bleeding Weakness Dyspepsia	0.20
	*Xanthium spinosum* L. 3461-KASH	Lokut-cxeer	N	N	Y	N	H	RT RT RT RT	Decoction Paste Paste Paste	Oral Topical Topical Topical	Fever Headache Wounds Abdominal pain	0.11
	*Xanthium strumarium* L. 3462-KASH	Cxeer	N	N	Y	N	H	RT RT FL FL	Decoction Decoction Decoction Decoction	Topical Topical Topical Topical	Boils Itching Sun burns Toothache	0.12
Balsaminaceae	*Impatiens glandulifera* Royle 2989-KASH	Goj-gassh	N	N	N	Y	H	WP WP LF LF	Paste Paste Infusion Decoction	Topical Topical Topical Topical	Sun burns Wounds Skin allergy Joint pain	0.08
	*Impatiens brachycentra* Kar. and Kir. 3402-KASH	-	Y	N	N	Y	H	FL SD FL LF	Infusion Powder Paste Infusion	Oral Topical Topical Oral	Tonic Snakebite Burns Aphrodisiac	0.29
Berberidaceae	*Berberis lycium* Royle 2970-KASH	Kawdach	Y	N	N	Y	S	LF FR FR	Paste Infusion Infusion	Topical Oral Oral	Toothache Constipation Diarrhea	0.26
	*Podophyllum hexandrum* Royle 3429-KASH	Wanwangun	Y	Y	Y	Y	H	RT RT	Decoction Decoction	Oral Oral	Diarrhea Body weakness	0.17
Brassicaceae	*Capsella bursa-pastoris* (L.) Medik 2971-KASH	Kralmond	N	N	Y	Y	H	LF LF LF	Cooked Decoction Decoction	Oral Oral Oral	Bleeding after delivery Vomiting Intestinal infection	0.30
	*Lepidium apetallum* L. 3409-KASH	Kulhaakh	N	N	Y	Y	H	LF LF AP AP	Decoction Decoction Infusion Paste	Oral Oral Oral Topical	Asthma Cough Tonic Fever	0.28
	*Lepidium didymum* L. 3410-KASH	Jangli-Halian	Y	N	Y	Y	H	WP LF WP	Poultice Power Paste	Topical Oral Topical	Fracture Vomiting Rheumatism	0.21
	*Nasturtium officinale* W.T. Aiton 3419-KASH	Kulhaakh	Y	Y	N	N	H	LF LF LF	Cooked Cooked Decoction	Oral Oral Oral	Indigestion Intestinal worms Constipation	0.14
	*Sisymbrium loeselii* L. 3448-KASH	Tilgogul gassh	N	N	N	Y	H	LF LF LF AP	Cooked Decoction Infusion Infusion	Oral Oral Oral Topical	Tonic Stomachache Sore throat Chest congestion	0.13
Cannabaceae	*Cannabis sativa* L. 3376-KASH	Bhang	Y	Y	N	Y	H	LF LF LF LF LF	Paste Paste Paste Infusion Infusion	Topical Topical Topical Oral Oral	Joint pain Ear-ache Depression Diarrhea Intestinal worms	0.19
Caprifoliaceae	*Sambucus wightiana* Wall. 3001-KASH	Gandula	Y	N	N	N	H	FR LF RT	Infusion Infusion Infusion	Oral Oral Oral	Stomach pain Indigestion Diuretic	0.14
Chenopodiaceae	*Chenopodium album* L. 2972-KASH	Konh	N	N	N	Y	H	LF LF LF LF	Cooked Decoction Decoction Decoction	Oral Oral Oral Oral	Painful urination Constipation Laxative Diarrhea	0.20
	*Chenopodium foliosum* (Moench.) Asch. 3607-KASH	Konh	Y	Y	N	N	H	LF FR FR LF	Paste Paste Paste Cooked	Topical Topical Topical Oral	Cold Breath shortness Cough Indigestion	0.29
Clusiaceae	*Hypericum perforatum* L. 2988-KASH	Shin-chae	Y	Y	N	N	H	LF FL FL WP	Poultice Powder Powder Decoction	Topical Topical Topical Oral	Joint pain Sores Wounds Prolonged menstrual bleeding	0.14
Convolvulaceae	*Cuscuta europaea* L. 2977-KASH	Kuklipot	Y	N	N	Y	H	WP WP WP	Paste Paste Paste	Topical Topical Topical	Sunburn Chest congestion Breathing problems	0.08
	*Ipomea purpurea* (L.) Roth. 3617-KASH	Ishq-e-phechan	N	N	N	Y	C	SD SD SD	Infusion Infusion Decoction	Oral Oral Oral	Anthelminthic Diuretic Laxative	0.09
Cucurbitaceae	*Cucumis sativus* L. 2976-KASH	Laer	Y	N	Y	Y	C	FR FR	Paste Paste	Topical Topical	Skin cleanser Fever	0.17
Equisetaceae	*Equisetum arvense* L. 2981-KASH	Bandakey	Y	N	Y	N	H	WP WP WP WP WP	Paste Paste Paste Infusion Infusion	Topical Topical Topical Oral Oral	Skin allergy Itching Strengthening of bones Diabetes Urinary disorder	0.17
Fabaceae	*Astragalus grahamianus* Benth. 3603-KASH	Zand posh	N	Y	N	N	S	RT RT RT	Decoction Decoction Decoction	Oral Oral Oral	Cold Cough Chronic bronchitis	0.10
	*Medicago polymorpha* L. 3625-KASH	Burahang	N	Y	N	Y	H	FL FL LF LF	Infusion Infusion Paste Paste	Oral Oral Topical Topical	Morning sickness Jaundice Pneumonia Chest congestion	0.14
	*Melilotus albus* Medik. 3413-KASH	Janglimethi	Y	Y	Y	N	H	WP LF LF	Paste Paste Powder	Topical Topical Topical	Fever Muscle pain Cuts	0.13
	*Robinia pseudoacacia* L. 2998-KASH	Kikar	Y	N	N	N	H	LF FL FL FL	Decoction Poultice Paste Paste	Topical Topical Topical Topical	Wounds Joint pain Fever Chilblain	0.19
	*Trifolium repens* L. 3455-KASH	Batak neeg	Y	N	N	Y	H	LF WP LF LF	Infusion Decoction Infusion Decoction	Oral Oral Oral Topical	Dry cough Debility Leucorrhea Gout	0.11
	*Trigonella foenum-graecum* L. 3456-KASH	Meth	Y	Y	Y	Y	H	SD LF	Decoction Decoction	Oral Oral	Indigestion Sore throat	0.20
Geraniaceae	*Erodium cicutarium* (L.) L’Her.ex Aiton 3393-KASH	Painzungajj	N	N	Y	Y	H	LF WP	Powder Paste	Oral Topical	Post-partum hemorrhage Headache	0.12
	*Geranium pratense* L. 2985-KASH	Ringrish	Y	N	Y	N	H	WP LF LF	Paste Infusion Infusion	Topical Oral Oral	Toothache Diarrhea Dysentery	0.17
	*Geranium wallichianum* Oliv. 2986-KASH	Ratanjoth	Y	N	Y	N	H	RT RT LF	Paste Paste Poultice	Topical Oral Topical	Wound antiseptic Fever Joint pain	0.19
Hippocastanaceae	*Aesculus indica* (Wall. eEx Jacquem) Hook. f. 3355-KASH	Handoon	N	N	N	Y	T	SD SD LF LF	Oil Oil Infusion Infusion	Topical Topical Oral Oral	Joint pain Cracked heals Cough Cold	0.12
Juglandaceae	*Juglans regia* L. 3405-KASH	Doon	Y	N	Y	Y	T	BR BR BR	Powder Poultice Paste	Topical Topical Topical	Toothache Wounds Skin rashes	0.23
Lamiaceae	*Ajuga bracteosa* Wall. ex Benth. 3356-KASH	Jani-adam	Y	Y	Y	N	H	WP WP	Infusion Infusion	Oral Oral	Abdominal pain Diarrhea	0.16
	*Ajuga parviflora* L. 3601-KASH	Jani-adam	Y	Y	Y	N	H	LF LF LF	Infusion Infusion Infusion	Oral Oral Oral	Abdominal pain Intestinal infection Kidney infection	0.23
	*Clinopodium umbrosum* (M.Bieb.) 3382-KASH	Kunakul	Y	N	Y	N	H	WP AP AP	Infusion Cocked Decoction	Topical Oral Oral	Astringent Tonic Carminative	0.11
	*Isodon rugosus* Wall. ex Benth. 3404-KASH	Maldah	N	N	Y	Y	H	LF LF LF LF	Paste Paste Powder Decoction	Topical Topical Topical Oral	Insect bite Abdominal pain Snake bite Vermifuge	0.17
	*Mentha aquatica* L. 3416-KASH	Kul pudni	Y	Y	Y	N	H	LF LF LF	Decoction Infusion Infusion	Oral Oral Oral	Influenza Abdominal cramps Induces sweating	0.14
	*Mentha arvensis* L. 3414-KASH	Pudni	Y	Y	Y	Y	H	LF LF	Decoction Decoction	Oral Oral	Stomach cramps Intestinal infection	0.16
	*Nepeta cataria* L. 2993-KASH	Brair-gassh	Y	N	N	N	H	LF LF	Paste Decoction	Topical Oral	Headache Fever	0.19
	*Prunella vulgaris* L. 2997-KASH	Kalweuth	Y	Y	N	N	H	FR FL FL	Decoction Paste Paste	Topical Topical Topical	Joint pain Headache Muscle pain	0.22
	*Stachys floccosa* Benth. 3645-KASH		N	Y	Y	N	H	WP WP	Decoction Infusion	Oral Oral	Amenorrhea Diuretic	0.21
Malvaceae	*Hibiscus syriacus* L. 3399-KASH	Jabakusam	N	Y	Y	N	S	FL FL LF	Decoction Infusion Infusion	Oral Oral Oral	Diuretic White discharge Body ache	0.10
	*Lavatera cashmiriana* Mast. 3408-KASH	Sazposh	Y	N	Y	Y	H	FL FL	Paste Paste	Topical Topical	Skin irritation Skin infection	0.22
	*Malva neglecta* Wall. 2991-KASH	Sochal	Y	Y	N	N	H	SD LF LF LF	Decoction Cooked Cooked Paste	Oral Oral Oral Topical	Fever Stomach cramps Body weakness Wounds	0.19
	*Malva sylvestris* L. 2992-KASH	Gur-sochal	Y	Y	N	N	H	LF LF	Paste Poultice	Topical Topical	Wounds Headache	0.17
Moraceae	*Ficus carica* L. 3395-KASH	Anjeer	N	N	N	Y	T	FR FR FR FR	Juice Juice Decoction Decoction	Oral Oral Oral Oral	Indigestion Body weakness Abdominal pain Lactation	0.20
	*Ficus palmata* Forssk. 3396-KASH	Anjeer	Y	Y	N	Y	T	LF FR FR LF	Decoction Juice Juice Infusion	Oral Oral Oral Topical	Stomach cramps Abdominal pain Urine infection Remove warts	0.19
Oxalidaceae	*Oxalis corniculata* L. 3423-KASH	Chuk-xanjj	N	N	N	Y	H	WP WP WP	Infusion Infusion Infusion	Oral Oral Oral	Abdominal pain Diarrhea Dysentery	0.25
Pinaceae	*Abies pindrow* (Royle ex D. Don) Royle 2965-KASH	Budul	Y	Y	N	N	T	LF LF LF LF	Paste Paste Paste Paste	Topical Topical Topical Topical	Skin rashes Cough Cold Toothache	0.16
	*Cedrus deodara* (Roxb. ex D. Don) G. Don. 3379-KASH	Deodar	Y	Y	Y	N	T	WD WD WD WD	Oil Oil Oil Oil	Topical Topical Topical Topical	Wounds Skin rashes Itching Joint pain	0.22
	*Pinus wallichiana* A. B. Jacks. 2994-KASH	Kayar	Y	Y	Y	N	T	ST ST	Oil Oil	Topical Topical	Skin rashes Boils	0.17
Plantaginaceae	*Plantago lanceolata* L. 2995-KASH	Gull	Y	N	Y	Y	H	LF LF LF LF	Tea Tea Tea Tea	Oral Oral Oral Oral	Cough Bronchitis Laxative Body weakness	0.22
	*Plantago major* L. 2996-KASH	Bed-Gull	N	N	N	Y	H	LF SD SD SD	Paste Poultice Poultice Decoction	Topical Topical Topical Oral	Skin rashes Bruises Rheumatic pain Urinary irritation	0.20
Poaceae	*Cynodon dactylon* (L.) Pers. 2979-KASH	Dramun	Y	Y	Y	N	H	WP WP WP	Paste Paste Poultice	Topical Topical Topical	Skin rashes Wounds Joint pain	0.11
	*Echinocola colona* (L.) Link 3391-KASH	Hamgass	N	N	N	Y	H	WP WP	Powder Paste	Topical Topical	Wound healing Body pain	0.09
	*Poa pratensis* L. 3632-KASH	Gass	Y	N	Y	N	H	SD WP	Cooked Powder	Oral Topical	Tonic Wound healing	0.10
Polygonaceae	*Bistorta amplexicaulis* (D.Don) Greene 3424-KASH	Marhan-chai	Y	Y	Y	N	H	RT RT RT RT	Paste Infusion Infusion Powder	Topical Topical Topical Topical	Headache Cold Cough Burns	0.18
	*Polygonum aviculare* L. 3430-KASH	Bamalia	Y	N	Y	N	H	LF LF WP	Infusion Infusion Infusion	Oral Oral Topical	Urinary tract infection Diuretic Boils	0.16
	*Rumex nepalensis* Spreng. 2999-KASH	Abijj	Y	Y	Y	N	H	RT RT LF	Juice Juice Paste	Topical Topical Topical	Headache Cuts Sores	0.19
Pteridaceae	*Adiantum capillus-veneris* L. 3354-KASH	Gewtheer	Y	Y	N	N	H	LF LF LF LF	Paste Paste Paste Paste	Topical Topical Topical Topical	Chest congestion Chest pain Asthma Headache	0.20
Rosaceae	*Cyndonia oblonga* Mill. 2978-KASH	Bumchoont	Y	Y	Y	N	T	SD FR FR	Decoction Juice Juice	Oral Oral Oral	Constipation Body weakness Antispasmodic	0.16
	*Geum roylei* Wall. ex. F. Bolle 2987-KASH		Y	N	Y	N	H	WP WP WP	Paste Paste Paste	Topical Topical Topical	Nasal congestion Skin allergy Breathing problems	0.09
	*Rosa indica* L.	Gulab	Y	Y	Y	Y	H	FL FL FL FL	Juice Juice Powder Paste	Oral Oral Oral Topical	Blood purification Throat ulcers Cough Anti-inflammatory	0.21
Rubiaceae	*Gallium aparine* L. 2984-KASH	Thapeh-gassh	Y	N	Y	N	H	LF LF WP	Paste Paste Paste	Topical Topical Topical	Wound antiseptic Skin allergy Diuretic	0.11
Salicaceae	*Salix alba* L. 3000-KASH	Veer	Y	N	N	Y	T	LF BR BR	Decoction Infusion Infusion	Topical Oral Oral	Joint pain Anthelminthic Headache	0.14
Scrophulariaceae	*Verbascum thapsus* L. 3458-KASH	Wantamook	Y	N	N	N	H	LF LF	Paste Paste	Topical Topical	Ear pus Burns	0.17
Solanaceae	*Datura stramonium* L. 2980-KASH	Datur	Y	N	Y	N	H	SD SD LF LF	Paste Powder Paste Paste	Topical Oral Topical Topical	Arthritic pain Cough Boils Burns	0.20
	*Solanum nigrum* L. 3002-KASH	Kambai	N	N	Y	Y	H	FR FR FR	Paste Paste Paste	Topical Topical Topical	Skin rashes Cold Cough	0.17
	*Solanum tuberosum* L. 3451-KASH	Alua	N	N	N	Y	H	TB TB TB	Cooked Paste Paste	Oral Topical Topical	Acidity Blisters Wounds	0.11
Urticaceae	*Urtica dioica* L. 3006-KASH	Soi	N	N	Y	Y	H	LF LF RT	Paste Paste Poultice	Topical Topical Topical	Wounds Skin infections Joint pain	0.12
Violaceae	*Viola odorata* L. 3007-KASH	Palfort	Y	Y	Y	Y	H	FL FL FL FL	Infusion Paste Paste Infusion	Oral Oral Oral Oral	Sore throat Chest congestion Bronchitis Cough	0.14
Vitaceae	*Vitis vinifera* L. 3008-KASH	Daech	Y	N	Y	Y	C	LF FR FR FR	Poultice Juice Juice Juice	Topical Oral Oral Oral	Sores Fever Jaundice Body weakness	0.16

**Abrreviations:** LF—leaf; RT—root; RH—rhizome; FL—flower; SD—seed; FR—fruit; WP—whole plant; TB—tuber; WD—wood; BR—bark; ST—stem: H—herb; S—shrub; T—tree; C—climber: Y—yes; N—no.

## Data Availability

The data used to support the findings of this study are available from the corresponding author upon request.

## Abbreviations

WHO—World Health Organization; IPNI—International Plant Names Index; KASH—Herbarium acronym; UV—use value; LF—leaf; RT—root; RH—rhizome; FL—flower; SD—seed; FR—fruit; WP—whole plant; TB—tuber; WD—wood; BR—bark; ST—stem: H—herb; S—shrub; T—tree; C—climber: Y—yes; N—no.

## Appendix A. Questionnaire

Name of the participant.
Participant’s age and gender.
Address of the participant.
Educational qualification of the participant.
Interview date.
How long do you live in the given area?
Local name of the used plant.
Which diseases are treated by the plant?
Which part is used?
What is the method of remedy preparation?
What is the approximate dose?
How long should a patient be using the plant?
Are there any possible side effects when one uses of the plant, or specific groups (e.g., children, pregnant women) who have to be careful or should not use it?
